# Eye-recognizable and repeatable biochemical flexible sensors using low angle-dependent photonic colloidal crystal hydrogel microbeads

**DOI:** 10.1038/s41598-019-53499-2

**Published:** 2019-11-19

**Authors:** Mio Tsuchiya, Yuta Kurashina, Hiroaki Onoe

**Affiliations:** 1 Graduate School of Integrated Design Engineering, Keio University, 3-14-1 Hiyoshi, Kohoku-Ku, Yokohama 223-8522 Japan; 2 Department of Mechanical Engineering, Faculty of Science and Technology, Keio University, 3-14-1 Hiyoshi, Kohoku-Ku, Yokohama 223-8522 Japan; 3 School of Materials and Chemical Technology, Tokyo Institute of Technology, 4259 Nagatsutacho, Midori-Ku, Yokohama 226-8503 Japan

**Keywords:** Biomedical engineering, Materials for devices

## Abstract

This paper presents eye-recognizable and repeatable biochemical flexible sensors using low angle-dependent stimuli-responsive photonic colloidal crystal hydrogel (PCCG) microbeads. Thanks to the stimuli-responsive PCCG microbeads exhibiting structural color, users can obtain sensing information without depending on the viewing angle and the mechanical deformation of the flexible sensor. Temperature-responsive PCCG microbeads and ethanol-responsive PCCG microbeads were fabricated from a pre-gel solution of *N*-isopropylacrylamide (NIPAM) and *N*-methylolacrylamide (NMAM) by using a centrifuge-based droplet shooting device (CDSD). As a proof-of-concept of thin and flexible biochemical sensors, temperature- and ethanol-sensing devices were demonstrated. By comparing the structural color of the stimuli-responsive PCCG microbeads and the color chart of the device, sensing information, including skin temperature of the human body and ethanol concentration in alcoholic beverages, was obtained successively. We expect that our device design using low angle-dependent stimuli-responsive PCCG microbeads would contribute to the development of user-friendly biochemical sensor devices for monitoring environmental and healthcare targets.

## Introduction

Stimuli-responsive hydrogels^1^ that shrink and swell in response to the intensity of external stimuli such as temperature, pH, and chemical substance have been applied in the field of drug delivery systems^2,3^, soft micro-actuators^4,5^, and biochemical sensors^6^. Particularly, sensors that respond to chemical and biological targets repeatedly for healthcare and environment sample monitoring have attracted growing interest. For applying these stimuli-responsive hydrogels for biochemical sensors, it is necessary to convert the intensity of the stimulus to another type of information. A photonic colloidal crystal hydrogel (PCCG), in which monodispersed colloidal particles with diameters of hundreds of nanometers are regularly arranged, appears to be a powerful tool for sensors based on a stimuli-responsive hydrogel^7–10^. PCCG that reflects visible-light wavelength through Bragg’s diffraction is called “structural color hydrogel”. In this hydrogel, the strength of the stimulus can be converted to visible color information by the change in the distance between the colloidal particles. Thus, it is possible to obtain the sensing information with the naked eye or by using simple optical devices such as a complementary metal-oxide-semiconductor camera.

By applying various functional polymers to PCCG, various eye-recognizable biochemical sensors for temperature^11^, humidity^12^, pH^13^, and certain analytes^14–17^ have been reported. For example, a PCCG that repeatedly responds to humidity was fabricated^12^. This composite hydrogel could be an economical alternative to traditional humidity sensors. These PCCG-based sensors have advantages such as simplicity in a measurement method, repeated use, non-fading color, and high efficiency in obtaining signals. However, as most conventional PCCG-based sensors are film-shaped, the peak wavelength and the color intensity change depending on the viewing angles, i.e., the so-called “angle dependency,” and on the mechanical deformation of the shape of PCCG^18,19^. This property makes it difficult to apply these hydrogels to practical sensors because the measurement result could differ depending on the viewing angle of the user and the mechanical deformation of the device.

**Figure 1:**
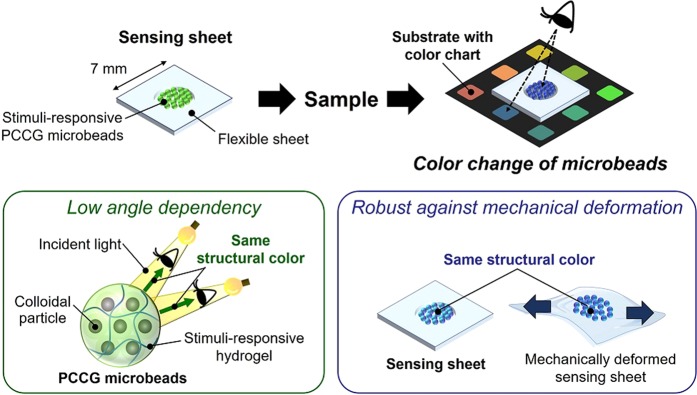
Schematic illustration of eye-recognizable and repeatable biochemical flexible sensors with stimuli-responsive PCCG microbeads. Users can obtain sensing information visually by the color of the microbeads. The freely dispersed stimuli-responsive PCCG microbeads suppress the change in the structural color against the viewing angle and the mechanical deformation.

Thus, we propose a stimuli-responsive PCCG-based eye-recognizable and repeatable biochemical flexible sensor with low angle dependency and high robustness against mechanical deformation by packaging stimuli-responsive PCCG microbeads as a sensing element (Fig. 1). By comparing the color of the stimuli-responsive PCCG microbeads with the color chart of the substrate by the naked eye, we can visually obtain the value of physical and chemical sensing information. Spherical colloidal photonic crystals have been applied in various fields such as a high throughput bio-screening in analytical chemistry and optical displays in device engineering thanks to its mass producibility, monodispersity and shape symmetry^20–22^. In this study, however, we proposed the use of photonic crystal microbeads to practical eye-recognizable biochemical sensors to suppress the change in the structural color against the observation angle and the unexpected mechanical deformation. Owing to the stimuli-responsive PCCG microbeads in the flexible sheet, the angle dependency of our sensors can be suppressed because the ordered arrangement of spatially symmetric photonic colloidal crystals (PCC) such as microspheres or hemispherical domes does not change depending on the viewing angle^23–26^. Moreover, as the stimuli-responsive PCCG microbeads are freely dispersed in the chamber formed on the flexible sheet, they can avoid unexpected color change with the mechanical deformation caused by the bending or stretching of the flexible sensing device. In this study, we used a “centrifuge-based droplet shooting device (CDSD)” for fabricating PCCG microbeads^27–29^. This method makes it possible to fabricate hydrogel microbeads easily and rapidly in a centrifugal separator, without loss of the pre-gel solution of stimuli-responsive polymers. As a demonstration of our proof-of-concept, we prepared and characterized two types of stimuli-responsive PCCG microbeads: a temperature-responsive sensor with poly(*N*-isopropylacrylamide) (PNIPAM) microbeads, and an ethanol-responsive sensor with poly(*N*-methylolacrylamide) (PNMAM) microbeads. Finally, we integrated these stimuli-responsive PCCG microbeads with thin and flexible devices to demonstrate the measurement of the skin temperature of a human hand and the ethanol concentrations of alcoholic beverages.

## Temperature-Responsive Microbeads

**Figure 2:**
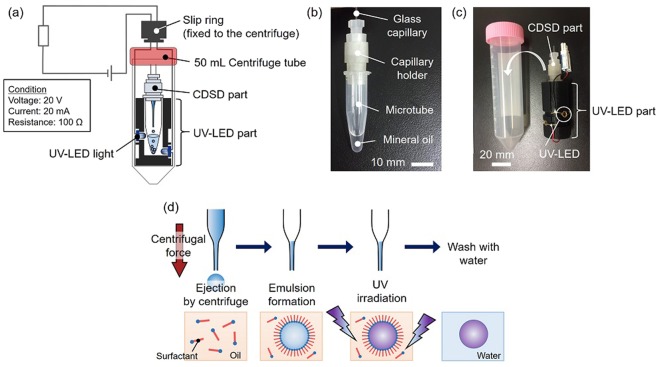
Fabrication of microbeads. (**a**) Schematic of the device setup for the fabrication of microbeads. (**b**) Setup of the CDSD part. (**c**) The assembly of the CDSD and the UV-LED parts and a 50 mL centrifuge tube. (**d**) The fabrication process of stimuli-responsive hydrogel microbeads. The pre-gel solution ejected by centrifugal force forms emulsion droplets in oil, which are polymerized by UV.

As an eye-recognizable biochemical sensor element, stimuli-responsive PCCG microbeads with the diameter of a few hundred micrometers are required so that they can be packaged in a thin and flexible device. We chose the CDSD-based method^27–29^ for obtaining monodispersed microbeads easily in a short time without sample loss. A setup for fabricating microbeads (Fig. 2a) was mainly composed of a CDSD part (Fig. 2b) and an ultraviolet light-emitting diode (UV-LED) part. The CDSD part was composed of a glass capillary (G-1, Narishige) filled with a pre-gel solution (Fig. S1), a lab-made capillary holder, and a 1.5 mL microtube. The UV-LED part has four UV-LED lights (UF3VL-1H411, DOWA Electronics Materials) with each radiant flux is 0.9 mW. An assembly of the CDSD and the UV-LED parts was set in a 50 mL centrifuge tube for centrifugation (H-19α, Kokusan) (Fig. 2c). To confirm whether the CDSD-based method could be applied to the stimuli-responsive hydrogel, we first tested the fabrication of stimuli-responsive microbeads without colloidal particles. In this process, the pre-gel solution was ejected from the tip of the glass capillary by a centrifugal force (~45 G) for 30–40 s, and a W/O emulsion was formed in the oil. Subsequently, the emulsion droplet was polymerized by UV and was washed several times with deionized (DI) water to remove oil (Fig. 2d).

**Figure 3:**
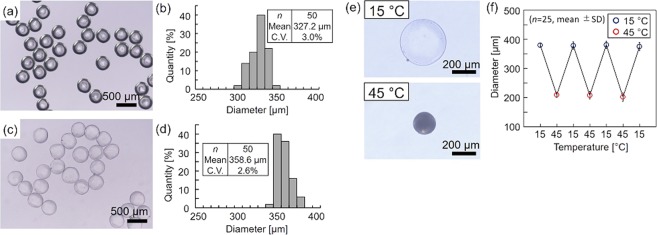
PNIPAM microbeads. Microscopic images and size distributions of PNIPAM microbeads (**a**,**b**) before washing and (**c**,**d**) after washing. (**e**) Microscopic images of PNIPAM microbeads at 15 °C and 45 °C. (**f**) Reversibility of the diameter change of PNIPAM microbeads.

Figure 3a–d show the microscopic images and size distribution of the fabricated PNIPAM microbeads before washing (in mineral oil) and after washing (in water). The diameters of the fabricated microbeads before and after washing were 327.2 µm ± 10.0 µm (mean ± s.d., *n* = 50) and 358.6 µm ± 9.3 µm (mean ± s.d., *n* = 50), respectively, ensuring that the microbeads were monodisperse (C.V. ≤ 3.0%). As the shape of the microbeads was maintained even after washing with water, the generated microdroplets of the pre-gel solution were properly polymerized by the UV irradiation in the centrifuge. We observed the difference in the diameters of the microbeads before and after washing with water (swell ratio: 9.6%). This result indicates that the hydrogel microbeads polymerized in oil had swollen by absorbing more water molecules in the hydrogel network after washing. In the usual fabrication system with the CDSD, the diameter of the ejected droplet, *d*, changes according to Tate’s law^27^ (*d* $\propto \sqrt[3]{{d}_{0}/G}$, where *d*_0_ is the tip of the capillary and *G* is the applied centrifugal force); however, in this method, it is also necessary to consider the diameter change by polymerization and swelling by washing to fabricate microbeads of the desired diameter.

The PNIPAM microbeads at both 15 °C and 45 °C exhibited a clear volume change (Fig. 3e) similar to that of conventional PNIPAM bulk hydrogel^30^. The PNIPAM microbeads also showed a reversible volume change with cyclic cooling and heating (Fig. 3f). These results show that functional stimuli-responsive hydrogel microbeads can be fabricated from the pre-gel solution through the CDSD-based fabrication method and that these fabricated microbeads could be applied to biochemical sensors for repeat uses as both the swelling degree and shrinkage degree were almost constant even if the temperature was changed repeatedly.

## Temperature-Responsive Structural Color Microbeads

To visualize the volume change of the stimuli-responsive hydrogel microparticles, PCC was simultaneously formed inside the hydrogel microbeads during the fabrication process to obtain the PCCG microbeads. PCCG exhibits structural color by immobilizing regularly arranged desalted colloidal particles with the hydrogel network. The peak reflective wavelength (*λ*_max_) of stimuli-responsive PCCG is expressed as

1 $${\lambda }_{{\rm{\max }}}\propto D\times \,\sin \,\theta \times C$$

where *D* is a distance between the colloidal particles, *θ* is the angle of the incident light, and *C* is a diameter change ratio (*C* = *d*/*d*_0_, where *d*_0_ and *d* are the diameters before and after the response, respectively)^31,32^. Thus, the change in *λ*_max_ for the stimuli-responsive PCCG before and after the response can be simply expressed as

2 $${\lambda }_{{\rm{\max }},{\rm{after}}}={\lambda }_{{\rm{\max }},{\rm{before}}}\times C.$$

As shown in Eqs (1) and (2), the peak wavelength of *λ*_max_ or *λ*_max,after_ shifts to the longer wavelength when the hydrogel swells (*C* > 1), and shifts to the shorter wavelength when the hydrogel shrinks (*C* < 1). Consequently, the visible structural color of the stimuli-responsive PCCG changes between the red side and the purple side owing to this characteristic. In stimuli-responsive hydrogels, the hydrogel volume changes according to the strength of the stimulus. Thus, the stimuli-responsive PCCG can convert the strength of the stimulus to the visible color change information.

**Figure 4:**
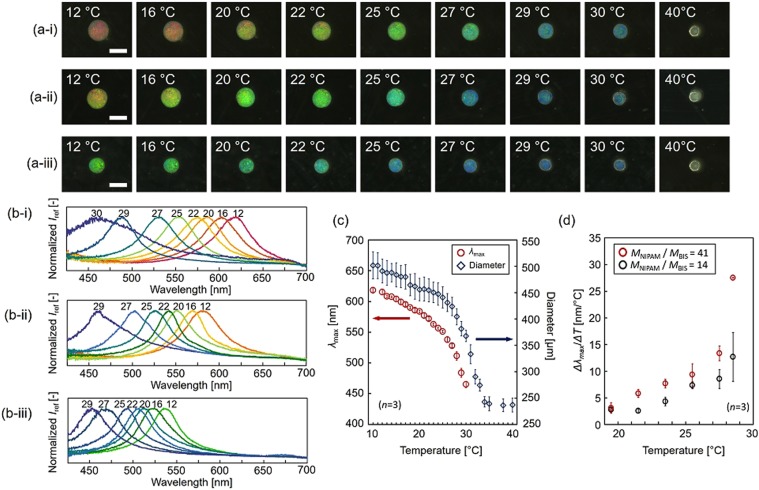
PNIPAM PCCG microbeads with different crosslinking ratio. (**a**,**b**) Microscopic images and reflection spectra of PNIPAM PCCG microbeads in DI water from 10 °C to 40 °C. (**a-i**,**b-i**) *M*_NIPAM_/*M*_BIS_ = 41, (**a-ii**,**b-ii**) *M*_NIPAM_/*M*_BIS_ = 27 and (**a-iii**,**b-iii**) *M*_NIPAM_/*M*_BIS_ = 14. Scale bars are 500 µm. *I*_ref_ shows the reflected light intensity. (**c**) Temperature dependence of the peak wavelength *λ*_max_ and the diameter of PNIPAM PCCG microbeads (*M*_NIPAM_/*M*_BIS_ = 41). (**d**) Comparison of the temperature dependence of Δ*λ*_max_/Δ*T* in PNIPAM PCCG microbeads for *M*_NIPAM_/*M*_BIS_ = 41 and 14.

For fabricating temperature-responsive PCCG microbeads, we prepared three types of pre-gel solutions with 130 nm SiO_2_ colloidal particles and with the different mole ratio of the monomer, *N*-isopropylacrylamide (NIPAM) to the crosslinker, *N*,*N*’-methylenebisacrylamide (BIS) (*M*_NIPAM_/*M*_BIS_ = 14, 27, and 41). Then, three types of temperature-responsive PCCG microbeads were fabricated with the CDSD method. Note that the centrifugal force does not cause the sedimentation or aggregation of SiO_2_ colloidal particles in the pre-gel solution (Figs S2, 3). The PNIPAM PCCG microbeads were observed by obtaining microscopic images in deionized water from 10 °C to 40 °C (Fig. 4a-i-iii). These images revealed that the PCCG microbeads exhibited a clear change in structural color from the red side to the purple side depending on the increase in temperature. In addition to the microscopic images, these PNIPAM PCCG microbeads were evaluated by measuring the reflection spectra with a UV-vis-NIR spectrometer (S4) under the same conditions (Fig. 4b-i-iii). These reflection spectra showed a clear peak wavelength at each temperature, and the total range of the shift of *λ*_max_ changed depending on the cross-linking ratio. This clear color change and *λ*_max_ change indicate that the fabricated PNIPAM PCCG microbeads can convert the stimulus information into color change information.

For confirming that the structural color changed owing to the volume change of the hydrogel as expressed by Eq. (3), we then compared the temperature dependence of the diameter and the temperature dependence of *λ*_max_ in the case of *M*_NIPAM_/*M*_BIS_ = 41 (Fig. 4c). Both the diameter and *λ*_max_ show similar curves that sharply changed around 32 °C, which is near the lower critical solution temperature (LCST) of PNIPAM^33^. Thus, this peak shift toward shorter wavelength caused by the temperature rise is considered to be due to the decrease in the particle distance caused by the shrinkage of the PNIPAM hydrogel. As the peak wavelength obtained through spectroscopic measurement, *λ*_max,meas_, and the peak wavelength calculated using Eq. (3), *λ*_max,calc_, (initial diameter and *λ*_max,before_ were set to be the values in the case of 10 °C, i.e., 618 nm and 502 µm, respectively) are reasonably close (*λ*_max,meas_ = 599 nm, and *λ*_max,calc_ = 596 nm at 16 °C, *λ*_max,meas_ = 557 nm, and *λ*_max,calc_ = 551 nm at 24 °C), these results show that PNIPAM PCCG microbeads changed isotopically in volume while maintaining the lattice structure (S4).

Furthermore, we evaluated the tuning of the sensitivity of the PNIPAM PCCG microbeads. As shown in Fig. 4a-i-b-iii, the total ranges of the shift of both the color and *λ*_max_ differ depending on the cross-linking ratio, *M*_NIPAM_/*M*_BIS_. For example, in the case of *M*_NIPAM_/*M*_BIS_ = 41, the color shift was large i.e., from red (615 nm, 12 °C) to purple (465 nm, 30 °C); in contrast, in the case of *M*_NIPAM_/*M*_BIS_ = 14, the color shift was smaller i.e., from yellow-green (538 nm, 12 °C) to purple (455 nm, 29 °C). Figure 4d shows the peak wavelength shift per temperature change (Δ*λ*_max_/Δ*T*), which indicates the sensitivity, in the case of *M*_NIPAM_/*M*_BIS_ = 41 and 14. As shown in Fig. 4d, Δ*λ*_max_/Δ*T* of *M*_NIPAM_/*M*_BIS_ = 41 was higher than that of *M*_NIPAM_/*M*_BIS_ = 14. This is because the hydrogel with lower cross-linking ratio swells and shrinks more easily, resulting in a larger change of the distance between the colloidal particles. This reveals that a lower cross-linking ratio makes the sensitivity higher. Therefore, the sensitivity of stimuli-responsive PCCG microbeads can be tuned by varying the crosslinking ratio.

**Figure 5:**
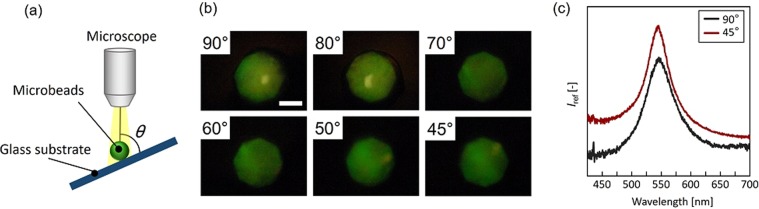
Measurement of angle dependency. (**a**) Setup of the measurement of angle dependency. (**b**) Microscopic images of PNIPAM PCCG microbeads from different observation angles. Scale bar is 200 µm. (**c**) Reflection spectra of PNIPAM PCCG microbeads observed from 0° and 45°.

An important feature of the PCCG microbeads is that their spherical shape can reduce the angle dependency of the PCCG. For confirming the low angle dependence of PNIPAM PCCG microbeads, we measured the reflection spectra of the PNIPAM PCCG microbeads by tilting the substrate from 45° to 90° (Fig. 5a). Microscopic images of the PCCG at varied observation angles, *θ*, (Fig. 5b) show that the dependence of the change in the structural color on the substrate angle was hardly observed. The reflection spectra measurement confirmed that no change in the peak wavelength was observed (90°: 545.8 nm, 45°: 545.8 nm) (Fig. 5c). This result suggests that the spherical structural color hydrogel is suitable for application to an eye-recognizable sensor.

## Stimuli-Responsive PCCG Microbeads for Sensing Chemicals

**Figure 6:**
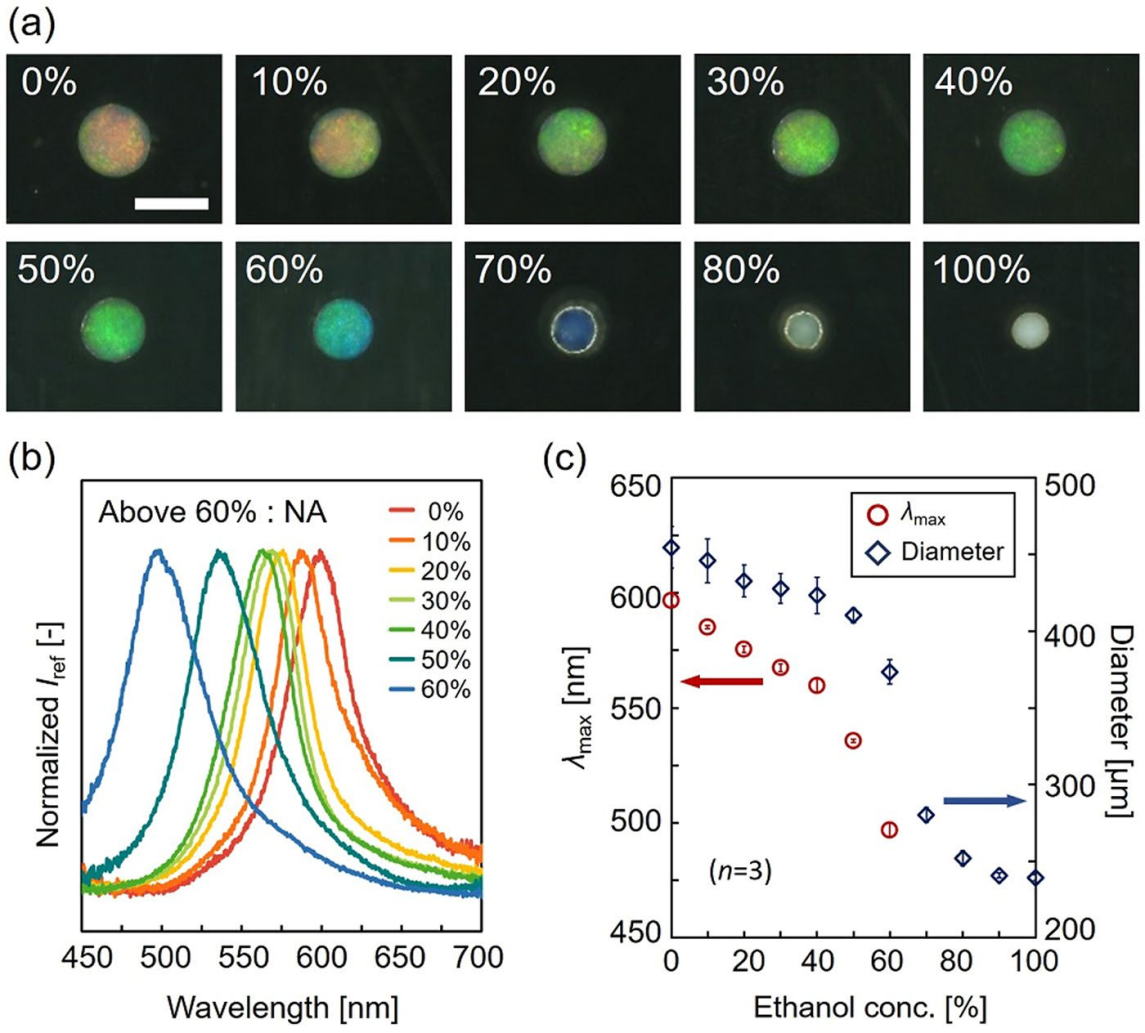
PNAM PCCG microbeads. (**a**) Microscopic images of PNMAM PCCG microbeads in the ethanol concentration from 0% to 100%. Scale bar is 500 µm. (**b**) Reflection spectra of PNMAM microbeads in the ethanol concentration from 0% to 60%. (**c**) Ethanol dependence of the peak wavelength *λ*_max_ and the diameter of PNMAM PCCG microbeads.

To demonstrate that this CDSD-based microbead fabrication method is applicable to various functional hydrogel polymers, we fabricated ethanol-responsive microbeads in the same way by using an ethanol-responsive monomer, *N*-methylolacrylamide (NMAM)^34^, and examined the ethanol responsivity. Although the pre-gel solution with 10% colloidal silica particle concentration is green in color, the fabricated microbeads exhibited a color closer to red (in the water). This is because the PNMAM hydrogel is more hydrophilic than the PNIPAM hydrogel, and is more likely to incorporate water molecules into the hydrogel network. Therefore, the final structural color of microbeads shifted to the longer-wavelength side through swelling. As the ethanol concentration increased from 0% to 100%, the color of the microbeads changed from red to the ultraviolet region while the diameter of the microbeads decreased (Fig. 6a). The reflection spectra measurement of the PNMAM PCCG microbeads confirmed that the peak wavelength shifted from the longer-wavelength side (597 nm) to the shorter-wavelength side (less than 497 nm) (Fig. 6b). The ethanol dependences of the diameter and *λ*_max_ of the microbeads are compared in Fig. 6c. Both the diameter and *λ*_max_ showed similar curves, indicating that this structural color change was due to the change in the distance of the colloidal particles caused by the volume change of the PNMAM hydrogel. From these results, we confirmed that the proposed microbead fabrication could be applied to other acrylamide derivatives that are polymerized with UV. The use of another acrylamide derivative monomer including vinyl-modified DNA aptamer makes it possible to prepare PCCG microbeads that respond only to a specific target such as specific low-molecular compounds^35^ and proteins^16^ without interfering with other targets.

## Eye-Recognizable and Flexible Sensing Devices

**Figure 7:**
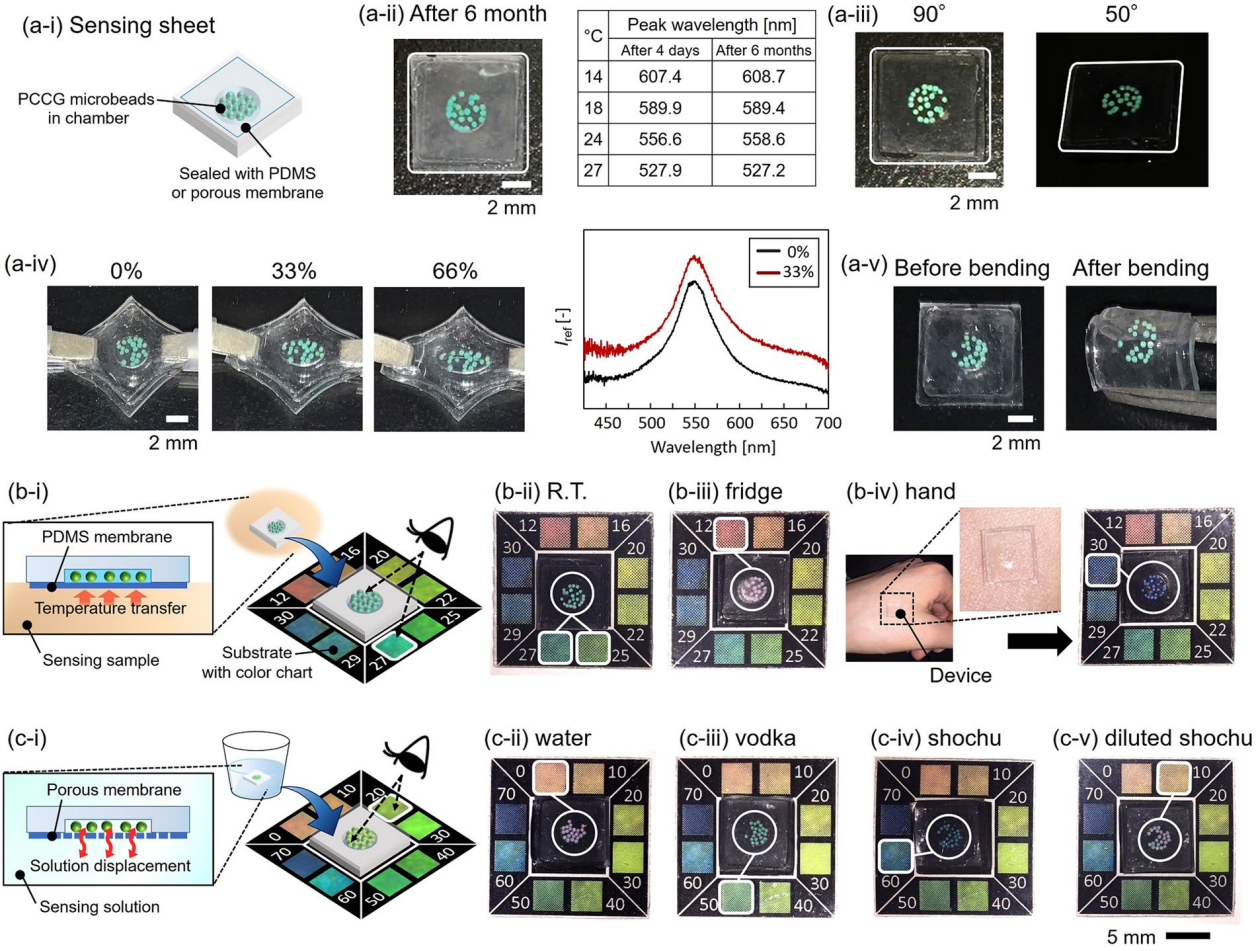
Eye-recognizable flexible sensing device. (**a-i**) Schematic illustration of the sensing sheet. Pictures of the flexible sheet (**a-ii**) 6 months after fabrication, (**a-iii**) viewing from 90° and 50°, (**a-iv**) stretching from 0% to 66% and (**a-v**) before/after stretching. (**b-i**) Schematic illustration explaining the working principle of the temperature sensing device. Pictures of the temperature sensing device after placed (**b-ii**) at the experimental desk, (**b-iii**) in the refrigerator and (**b-iv**) on the skin of the hand. (**c-i**) Schematic illustration explaining the working principle of the ethanol sensing device. Pictures of the ethanol-responsive sensing device after immersing (**c-ii**) in the water, (**c-iii**) in vodka (Alc. 50%), (**c-iv**) in shochu (Alc. 62%), and (**c-v**) in diluted shochu (Alc. 10%). Brightness has been modified for the visibility in device pictures.

Finally, we integrated these sensor microbeads with flexible devices to evaluate their measurement performances. Our sensing device was composed of a sensing sheet with stimuli-responsive PCCG microbeads and a substrate with a color chart. We built the sensing sheet by placing water-dispersed PCCG microbeads in the chamber (3 mm in diameter and 0.6–1.0 mm in-depth) on the flexible polydimethylsiloxane (PDMS) sheet (7 mm × 7 mm, thickness ~1.5 mm) and then sealed the PCCG microbeads with a lab-fabricated PDMS membrane (thickness ~70 µm) or a porous polycarbonate membrane (manually punched ~70 µm holes in commercially available porous polycarbonate membrane (TCTP02500, Millipore) with 10 µm pores) for temperature sensing and ethanol sensing, respectively (Figs 7a–i, S4). We first evaluated the stability, the low angle dependency and the robustness against the mechanical deformation of the sensing sheet. Even after 6 months, the structural color of the PNIPAM PCCG microbeads was clearly observed as long as the device was stored while preventing drying. The comparison between the PNIPAM PCCG microbeads 4days after the fabrication and the ones 6 months after the fabrication showed that they had almost the same change in the peak wavelength depending on the temperature change (Fig. 7a-ii). Moreover, the structural color of the PNIPAM PCCG microbeads was observed to be almost unchanged by the change in the viewing angles (*θ* = 90° and 50° in Fig. 7a-iii). To confirm the robustness against the mechanical deformation of the sensing sheet, we stretched the sensing sheet from 0% to 66% or bent it with tweezers (radius of the curvature: ~2 mm) (Fig. 7a-iv,v). We confirmed that the freely dispersed PNIPAM PCCG microbeads maintained its spherical shape and the structural color did not change even when the sensing sheet was deformed (*λ*_max, 0%_ = 549.4 nm, *λ*_max, 33%_ = 549.0 nm). Therefore, stretching or bending does not cause the unexpected color change of the PCCG microbeads. From these results, we confirmed that the microbead-shaped structural color sensing elements effectively contributed to the suppression of angle dependency of the exhibited structural colors and to the color robustness against the deformation of the entire device.

A schematic illustration (Fig. 7b-i) shows the working principle of the flexible temperature-sensing device using PNIPAM PCCG microbeads. The sensor microbeads sense the temperature of a sensing target via the PDMS membrane. We confirmed that the sensor microbeads could change its structural color within 5 min (Fig. S5a). After placing the flexible sensing sheet at a measurement point, the color of the PNIPAM PCCG microbeads can be converted into the temperature by comparing the color with the color chart. Temperature measurement has been demonstrated by placing the sensor at three different conditions successively: at the experiment desk in the office (~25.5 °C), in the refrigerator (~9 °C), and directly on the skin of a hand (~34 °C). On the experiment desk, the color of the microbeads indicated 25–26 °C, which was almost the value shown by a room-temperature meter, 25.5 °C (Fig. 7b-ii). In the refrigerator, the color of the microbeads changed to red (Fig. 7b-iii), which was similar to the color indicating 12 °C on the color chart. On the skin of the hand, the color of the microbeads changed to purple (Fig. 7b-iv), which was similar to the color indicating 30 °C on the color chart. These results show that temperature can be measured visually and repeatedly with this device.

Subsequently, the measurement of ethanol concentration was demonstrated by using three different alcoholic beverages: vodka (Russian distilled spirits, Alc. 50%), shochu (Japanese distilled spirits, Alc. 62%), and diluted shochu (Alc. 10%). In the case of the ethanol sensing, the flexible sensing sheet, where the water-dispersed NMAM PCCG microbeads were sealed in the chamber with the porous membrane, was immersed in a sample liquid and stirred for approximately 5 min to exchange the water in the chamber with the sample liquid completely via the porous membrane (Figs 7c-i, S5b). Before the measurement, the color of the NMAM PCCG microbeads was red (water: alcohol conc. = 0%); however, after immersing the sensing sheet in these alcoholic beverages in sequence, the color changed corresponding to each sample’s alcohol concentration (blue: ~60% conc., green: ~50% conc., and orange: ~10% conc. for vodka, shochu, and diluted shochu, respectively (Fig. 7c-ii-v)). These results show that our ethanol-sensing device has selectivity on different liquors if those liquors have different alcohol concentrations. Besides, the chemical substance, i.e., ethanol, can be measured visually and repeatedly with this device even if the sensing sample is not a pure ethanol solution but a mixture containing components other than ethanol such as alcoholic beverages.

## Discussion

One of the features of our proposed sensor with feely-dispersed stimuli-responsive PCCG microbeads is the robustness of the structural color against the deformation of the device. This device design avoids the unexpected structural color change caused by the bending or stretching of the device. Therefore, this sensor can be applied to the measurement of shape-deforming objects and thin flexible samples, such as biological skin surfaces and cloth surfaces, respectively, which cannot be measured with the conventional sheet-shaped PCCG because of the change in the structural color depending on the mechanical deformation of the device^18,19^.

From the viewpoint of materials, tuning and enhancing the functionality of the stimuli-responsive PCCG microbeads will lead to the improvement of the sensor performance and will broaden the range of its application. As mentioned earlier, the sensitivity of the PCCG microbeads can be tuned by varying the cross-linking ratio. In addition to the sensitivity, the measurement range could be controlled by copolymerizing the monomer material with another monomer material. For example, LCST control by copolymerizing another monomer with NIPAM has been reported^36,37^. LCST can be increased beyond 32 °C by increasing the content of a hydrophilic monomer such as *N*,*N*-dimethylacrylamide, and decreased below 32 °C by increasing the content of a hydrophobic monomer such as butyl methacrylate. Therefore, applying this LCST tuning to our PNIPAM PCCG microbeads facilitates application to more varied target measurements. Furthermore, the use of other stimuli-responsive materials such as molecularly imprinting polymers^38^ and DNA hydrogel^39,40^ in these PCCG microbeads could broaden the range of application for the detection of specific chemicals ranging from ions to macromolecules in the environment or healthcare monitoring.

However, as our sensor is designed for obtaining sensing information visually, it may not be suitable for users who need to obtain sensing information precisely as a numerical value, or for users with color weakness. This problem could be solved by adopting a camera system that can convert color information to an exact numerical value to support the readout of the color. Thus, a more precise and user-friendly sensor could be achieved using our stimuli-responsive PCCG-microbeads-based biochemical sensor.

We expect that our work can be applied to a more user-friendly environment and healthcare monitoring system with the advantageous features of PCCG-based sensors, such as repeated use and no requirement of external electrodes.

## Methods

### Materials

The colloidal silica solution containing amorphous silica particles with diameters of 130 nm (MP-1040) was provided by Nissan Chemicals. NIPAM (095-03692) and BIS (134-02352) were purchased from Wako Pure Chemical Industries. NMAM (M0574) was purchased from Tokyo Chemical Industry. Span 80 (37408-32) was purchased from Kanto Chemical. A photopolymerization initiator (Irgacure1173) was obtained from BASF. Mineral oil (M8410) was purchased from Sigma-Aldrich. Ion exchange resins (AG501-X8) were purchased from BIO-RAD. For sensor device fabrication, PDMS (SILPOT 184) was purchased from Dow Corning Toray, polycarbonate porous membrane (TCTP02500) was purchased from Millipore, and a polyethylene terephthalate (PET) film (TOYOBO ESTER®Film E5001) was purchased from Toyobo.

### Setup for fabricating microbeads

In the CDSD part, the glass capillary (G-1, Narishige), a lab-made capillary holder, and a 1.5 mL microtube were assembled. The tip of the glass capillary was sharpened with a puller to form a thin tip (PC-10, Narishige) and then cut out with a microforge (MF-900, Narishige) to the desired diameter (Fig. S1). The assembled CDSD was set in the UV-LED part. The UV-LED part is a jig made of polylactic acid using a 3D printer (Sigma, BCN3D Technologies) with four UV-LED lights (UF3VL-1H411, Dowa Electronics Materials) mounted around the jig. The UV-LED lights were connected to a DC power supply through a slip ring (Moog Components Group, EC4294-2) for supplying electric power to the centrifuge (H-19Rα, Kokusan).

### Microbeads fabrication

For the fabrication of microbeads without colloidal particles, a pre-gel solution composed of NIPAM (0.8 M), BIS (*M*_NIPAM_/*M*_BIS_ = 27), and a photopolymerization initiator (Irgacure1173) (0.5 vol%) was prepared. In the case of fabricating stimuli-responsive PCCG microbeads containing colloidal particles, a pre-gel solution composed of NIPAM (0.7 M), BIS (*M*_NIPAM_/*M*_BIS_ = 14, 27, 41), Irgacure1173 (0.5 vol%), and silica particles (final concentration 10 wt%) was prepared, and then desalted for more than 5 min with the ion exchange resin. In the case of PNMAM microbeads, we used NMAM (0.8 M NMAM, *M*_NMAM_/*M*_BIS_ = 46) instead of NIPAM. The pre-gel solution was loaded into the glass capillary, and the solution-filled capillary was fixed with the capillary holder to the 1.5 mL microtube containing mineral oil with Span 80 (3 wt%) and Irgacure1173 (0.5 vol%). The distance between the tip of the glass capillary and the air–oil interface was set to ~4 mm. The pre-gel solution was ejected via centrifugal force (~45 G) for 30–40 s, and a W/O emulsion was formed in the oil. Subsequently, the microtube was irradiated with UV (325 nm) in the centrifuge for 50 s for polymerizing the emulsion droplets. In these operations, the centrifuge was set at 5 °C for preventing shrinkage of the polymerized NIPAM owing to an increase in temperature. The polymerized hydrogel microparticles were washed several times with DI water to remove oil.

### Observation and evaluation of microbeads

The fabricated microbeads were observed by using an inverted phase-contrast microscope (IX73P1-22FL/PH, Olympus) and a digital microscope (VHX-100F, Keyence). The reflection spectra of the stimuli-responsive PCCG microbeads were obtained using an epi-illuminated microscope (BX-50, Olympus) equipped with a UV–vis–NIR spectrometer (USB2000+, Ocean Optics) and an Olympus U-LH100 light source. The angle dependence was observed by attaching a compact digital camera (EX-F1, CASIO) on the epi-illuminated microscope with an adapter (NY-F1, CASIO).

### Integration of stimuli-responsive PCCG microbeads with the flexible sensing device

For preparing the sensing sheet, the water-dispersed PCCG microbeads were placed in the chamber (3 mm in diameter and 1000 or 600 µm in-depth) fabricated on the PDMS sheet (7 mm × 7 mm, thickness ~1.5 mm) and sealed with the PDMS membrane or the porous membrane for the measurement of temperature or ethanol concentration, respectively (Fig. S4). We performed the demonstration of bending and stretching with the sensing sheet having the 1000 µm-depth chamber, and the other experiments were performed with the sensing sheet having the 600 µm-depth chamber. For preparing the substrate with the color chart, the colors of the microbeads were printed on a paper (18 mm × 18 mm), and then the PET film was affixed thereon for water-proofing.

To evaluate the measurement performance of the temperature-sensing device, the sensing sheet was first placed at the experiment desk in the office, then in the refrigerator, and finally on the skin of the hand. In each measurement, the sensing sheet was left for 5 min or more in the measurement condition. Then, for obtaining the measurement result, the sensing sheet was placed on the substrate to compare the color of the PCCG microbeads with the color chart.

For the evaluation of the measurement performance of the ethanol sensing device, three types of alcoholic beverages were used. The sensing sheet was first immersed in vodka (Smirnoff Vodka Blue 50°, Kirin), then in shochu (Yonaguni Miniature Kuba-Maki, Sakimoto Shuzo), and finally in diluted shochu (mixing ratio of shochu and DI water was 1:5). In each measurement, the sensing sheet was immersed in about 40 mL of alcoholic beverage with stirring for 5 min or more. Note that the minimum limit of the sample liquid depends on the size of the chamber and allowable error because our ethanol sensing device detects ethanol concentration by exchanging the water in the chamber with the sample liquid completely. Subsequently, the measurement results were obtained by placing the sensing sheet on the substrate for comparing the color of the PCCG microbeads with the color chart. The alcohol concentration of each beverage was also measured using a commercialized alcohol refractometer (Handheld Alcohol Meter, KKmoon).

## Supplementary information

Supplementary Information
