# Supplementary material for: Eye-recognizable and repeatable biochemical flexible sensors using low angle-dependent photonic colloidal crystal hydrogel microbeads
Source: Sci Rep. 2019 Nov 19;9:17059. doi: 10.1038/s41598-019-53499-2 (PMC6863886; doi:10.1038/s41598-019-53499-2)
Supplement: Supplementary file 1 — Supplementary Information [file 41598_2019_53499_MOESM1_ESM.docx]

**Supplementary Information**

**Eye-recognizable and repeatable biochemical flexible sensors using low angle-dependent photonic colloidal crystal hydrogel microbeads**

**Mio Tsuchiya^1^, Yuta Kurashina^2,3^, Hiroaki Onoe^*1^**

^1^ Graduate School of Integrated Design Engineering, Keio University 3-14-1 Hiyoshi, Kohoku-Ku, Yokohama, 223-8522, Japan, E-mail: onoe@mech.keio.ac.jp

^2^ Department of Mechanical Engineering, Faculty of Science and Technology, Keio University 3-14-1 Hiyoshi, Kohoku-Ku, Yokohama, 223-8522, Japan

^3^ School of Materials and Chemical Technology, Tokyo Institute of Technology 4259 Nagatsutacho, Midori-Ku, Yokohama, 226-8503, Japan.

Table of Contents

S1. Glass capillary for the CDSD (Figure S1)

S2. Effect of the centrifugal force to the pre-gel solution (Figure S2)

S3. SEM images of the PCCG microbeads (Figure S3)

S4. Calculation of the reflection spectra and λ_max,calc_ (Table S1)

S5. Details of the sensor design (Figure S4)

S6. Response time of the device (Figure S5)

S1. Glass capillary for the CDSD

The shape of the glass capillary differs whether the colloidal particles are mixed in the pre-gel solution or not. For the pre-gel solution without colloidal particles, as the viscosity of the pre-gel solution was relatively low, we used the glass capillary with a long narrow tip. Owing to the narrow tip, the ejection speed of the pre-gel solution can be moderated. We adjusted the diameter of the tip of the glass capillary 40 µm by the microforge. We inserted the pre-gel solution in this glass capillary by capillary action.

On the other hand, as the pre-gel solution with colloidal particles had relatively high viscosity, we used the glass capillary with a shorter narrow tip. We adjusted the diameter of the tip of the capillary 50-60 µm. We inserted the pre-gel solution into this glass capillary by pipetting 6 µL.


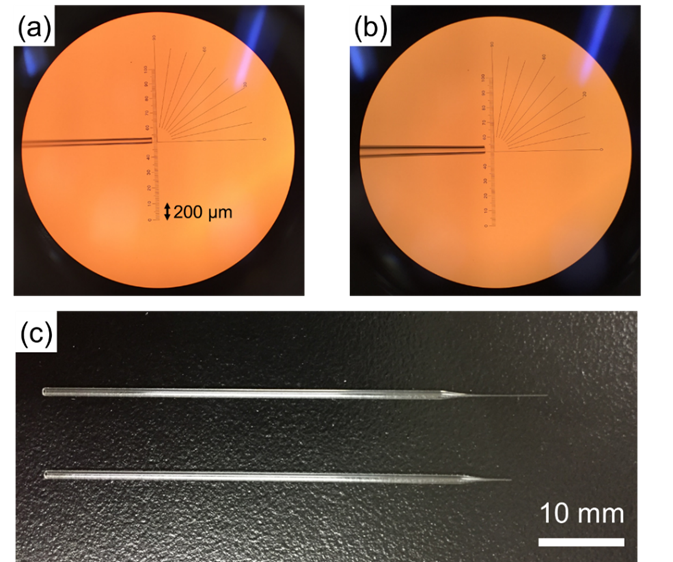


Figure S1 Fabricated glass capillaries. Enlarged view of the tip of the capillary for (**a**) the pre-gel solution without colloidal particles, (**b**) the pre-gel solution with colloidal particles. (**c**) Picture of the glass capillaries (upper: for the pre-gel solution without colloidal particles, lower: for the pre-gel solution with colloidal particles).

S2. Effect of the centrifugal force to the pre-gel solution

To check whether the centrifugal force causes the sedimentation or aggregation of SiO_2_ colloidal particles, we applied centrifugal force (~45 G) for 1 min. As Figure S2 shows, the centrifuged pre-gel solution showed clear and uniform structural color and did not show any sedimentation or aggregation of SiO_2_ colloidal particles in the pre-gel solution.


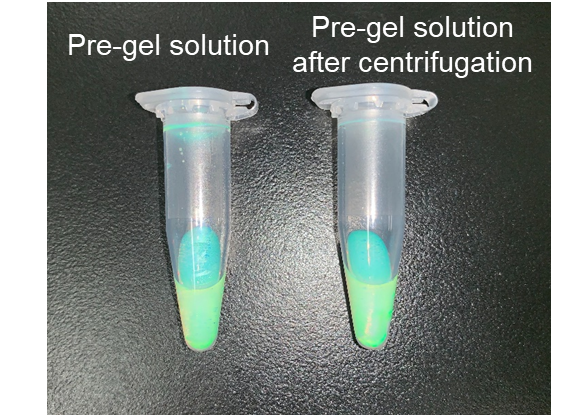


Figure S2 Pre-gel solution (left) and pre-gel solution after centrifugation (right).

S3. SEM images of the PCCG microbeads

To confirm whether the colloidal particles are regularly arranged in the PCCG microbeads, we obtained scanning electron microscopy (SEM) images with a field emission SEM (S-4700, Hitachi High-Technologies Group). Figures S3ab shows the PCCG microbeads fabricated by the pre-gel solution with 20% and 10% colloidal particles, respectively. When the concentration of colloidal crystal was 20%, we recognized a face-centered cubic (fcc) structure. On the other hand, when the concentration of colloidal crystal was 10%, we found the fcc structure not in an entire region of the microbeads but in some local domains.


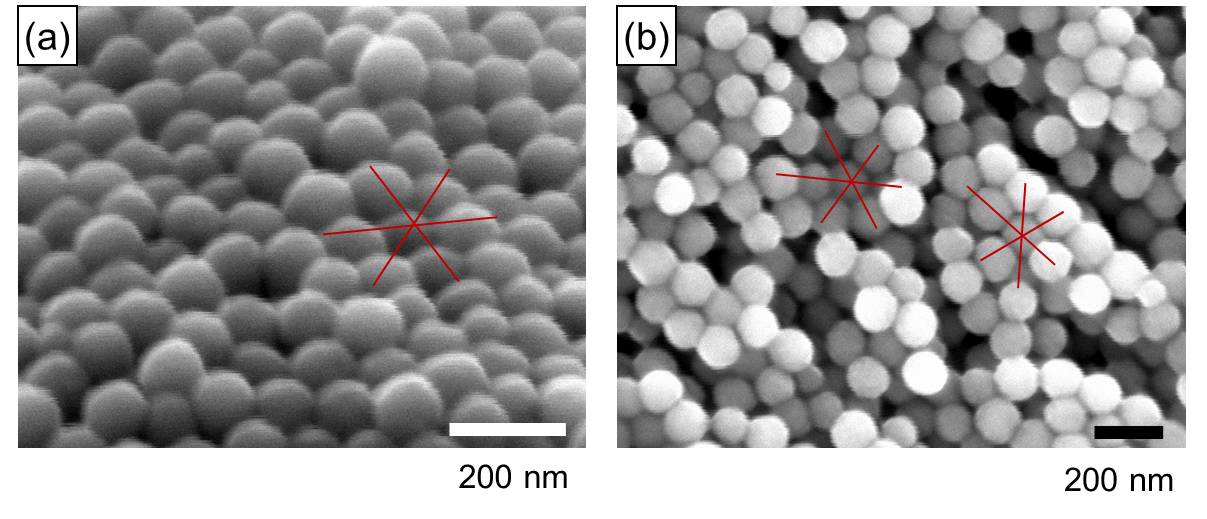


Figure S3 SEM images. (**a**) PNIPAM PCCG microbeads fabricated by the pre-gel solution with 20% colloidal particles, (**b**) PNMAM PCCG microbeads fabricated by the pre-gel solution with 10% colloidal particles.

S4. Calculation of the reflection spectra and λ_max,calc_

For obtaining reflection spectra of stimuli-responsive PCCG microbeads, we obtained three measurements: background signal intensity *I*_0_, total reflected light intensity *I*_m_ and stimuli-responsive PCCG microbeads’ reflected light intensity *I*_n_. Using these measurements, we calculated the reflected intensity *I*_ref_ as follows,

$\text{I}_{\text{ref}}\text{ = }\frac{\text{I}_{\text{n}}\text{-}\text{I}_{\text{0}}}{\text{I}_{\text{m}}\text{-}\text{I}_{\text{0}}}$ (S-1)

In the graph, we used the normalized *I*_ref_.

For comparing the peak wavelength obtained through spectroscopic measurement, *λ*_max,meas_, and the peak wavelength based on the diameter of the microbeads, *λ*_max,calc_, we calculated *λ*_max,calc_ using Eq.(S-2) and the following values: the diameter before response, *D*_0_ = 502 µm, the peak wavelength before response, *λ*_max,before_ = 618 nm.

*λ*_max,calc_ = *λ*_max,before_×*C* (S-2)


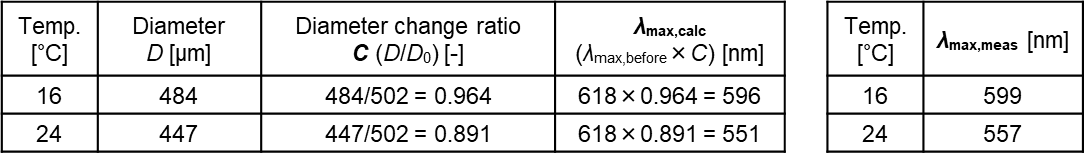
Table S1 Calculation of *λ*_max,calc_

S5. Details of the sensor design

The flexible sheet was formed of PDMS. The mixed material of the PDMS base material and the curing agent in an 18:1 or 10:1 ratio was poured on a lab-made acrylic mold, and then, cured on a hotplate at 75 °C for 1.5 h. After placing the water-dispersed stimuli-responsive PCCG microbeads in the chamber, the chamber was sealed with the PDMS membrane or the porous membrane by using uncured PDMS as an adhesive. For curing adhesive PDMS, the flexible sheet was stored at room temperature overnight. The fabrication process of the PDMS membrane and the porous membrane are shown below.

PDMS membrane: A glass slide was cleaned with acetone and covered with Optool (HD-1100TH, Daikin) as a release agent. The glass slide was placed on the hotplate at 75 °C for 5 min for drying Optool. Then, the glass slide was coated with the PDMS using a spin coater at 800 rpm for 60 s. The coated glass slide was placed on the hotplate at 75 °C for 1 h for curing. The thickness of the cured PDMS membrane was about 70 µm.

Porous membrane: The purchased porous membrane (pore size 10 µm) was manually punched with a suitable number of holes (diameter ~70 μm) before used for accelerating the liquid replacement in the chamber.


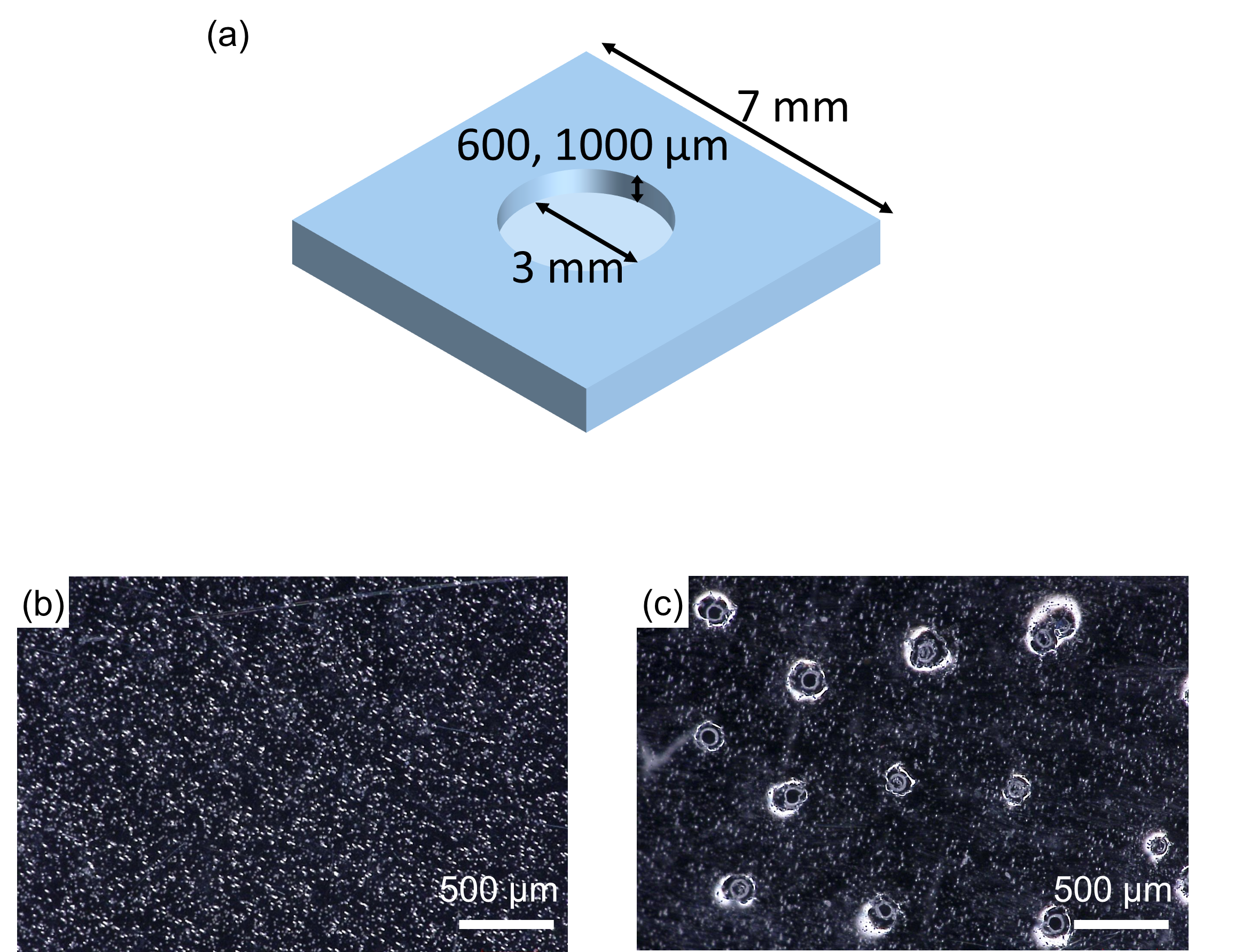


Figure S4 Details of the sensor design. (**a**) Image of the flexible sheet. Enlarged view of the porous membrane (**b**) before punching holes, (**c**) after punching holes.

S6. Response time of the device

We checked the response time of the sensor microbeads in our device by analyzing the change of the hue of the sensor microbeads over time. We observed the hue change of the sensor microbeads when the device was placed at from 9°C to 27°C for the temperature-sensing device, and when the device was immersed in the 60% ethanol solution from water, respectively. Then, we took the images of the sensor microbeads by a digital camera and analyzed the hue of the obtained images with ImageJ software. As Figure S5 shows, 5 min is enough for the sensor microbeads to respond to the sample in both temperature- and ethanol- sensing devices.


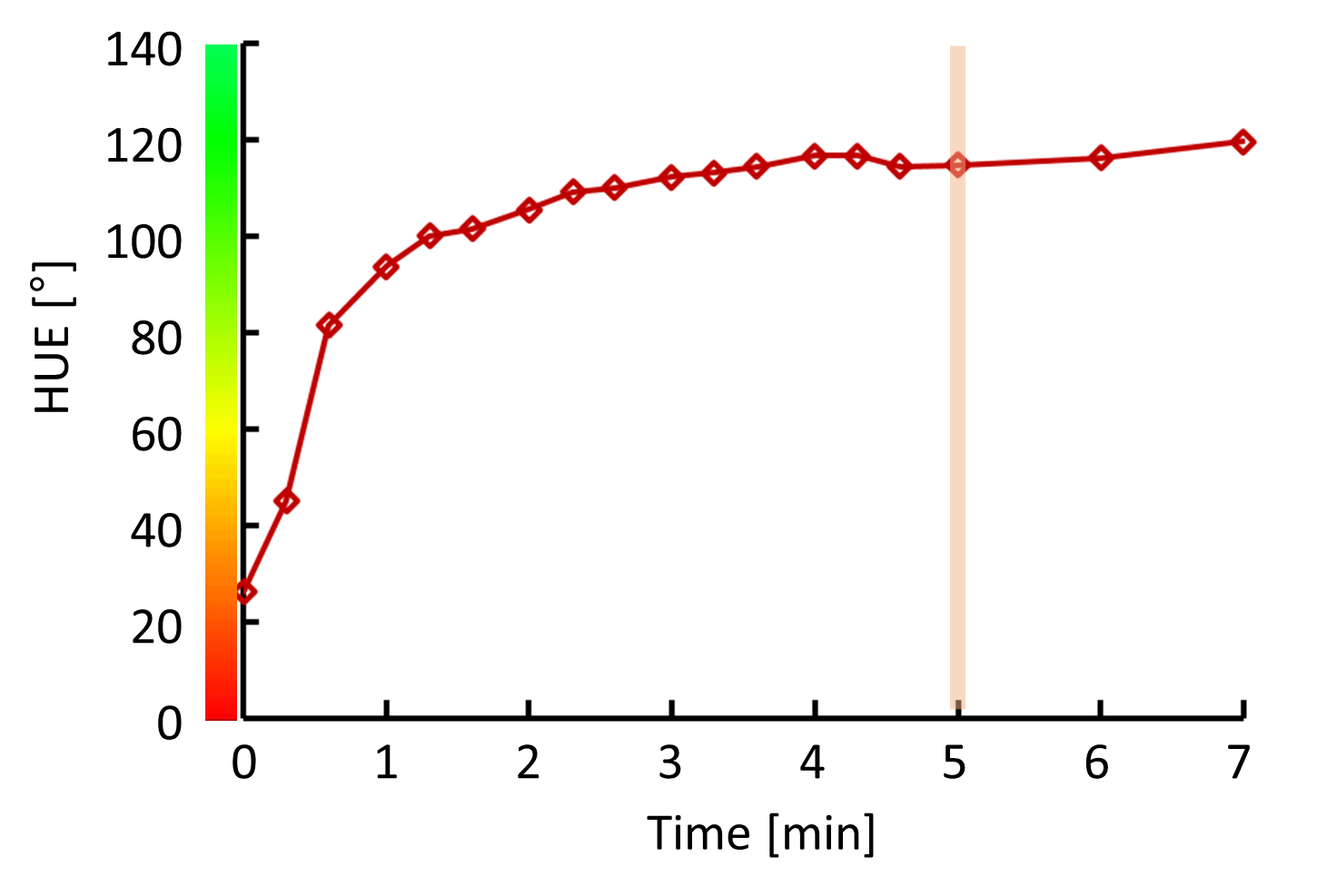

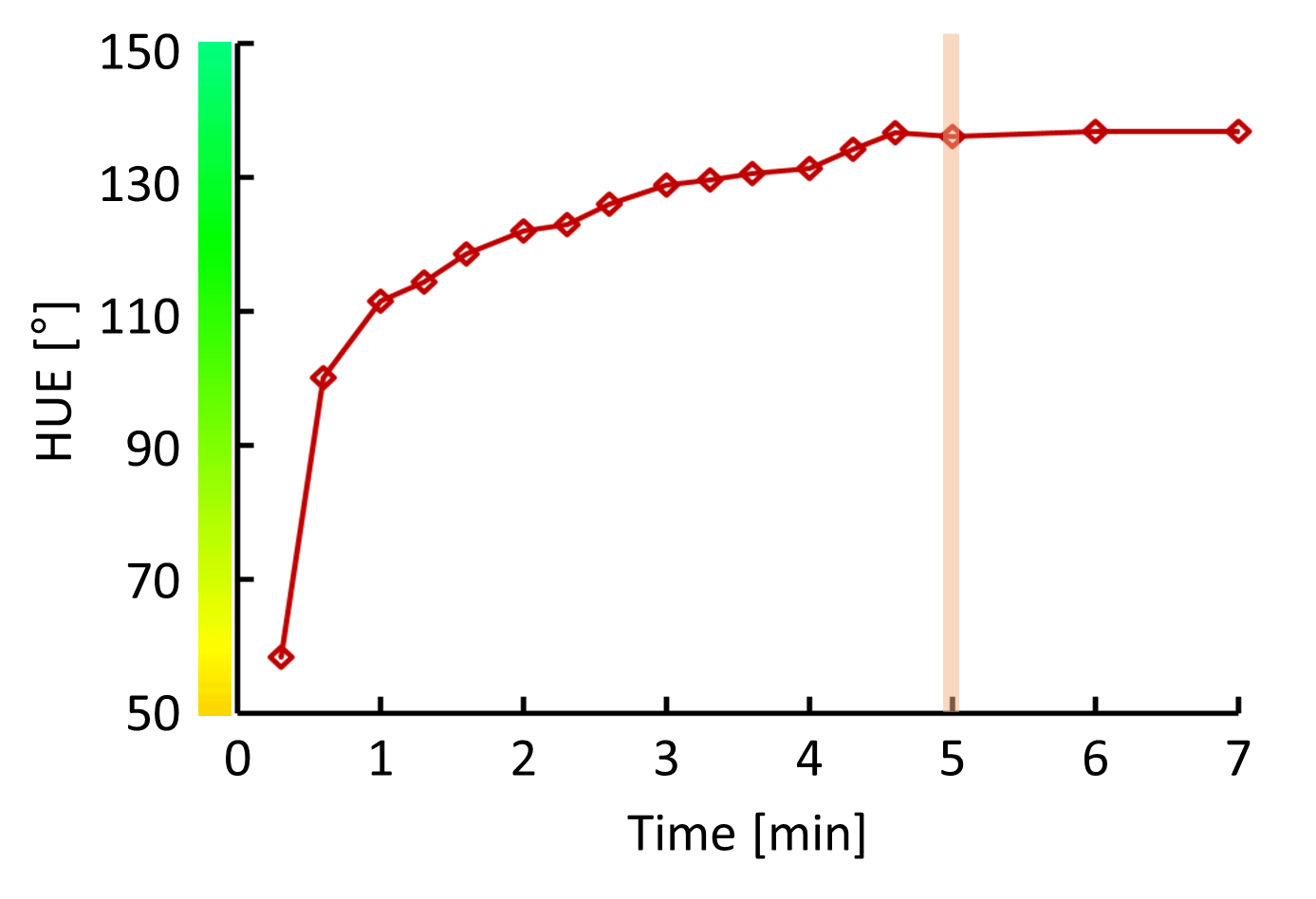


Figure S5 Change in the hue of the sensor microbeads over time. (**a**) temperature-sensing device. (**b**) ethanol-sensing device.
